# Clinical trial-related financial considerations from Deep South-patients with breast cancer who previously declined trial participation

**DOI:** 10.1007/s00520-025-09823-w

**Published:** 2025-08-09

**Authors:** Courtney P. Williams, Nicole Reh, Mandy Chen, Nicole Henderson, Nicole E. Caston, Gabrielle B. Rocque, Lily Gutnik

**Affiliations:** 1 Division of General Internal Medicine & Population Science, University of Alabama at Birmingham, 1720 2 Ave South, BDB 852, Birmingham, AL 35294 USA; 2 O’Neal Comprehensive Cancer Center, University of Alabama at Birmingham, Birmingham, AL USA; 3 Heersink School of Medicine, University of Alabama at Birmingham, Birmingham, AL USA; 4 Division of Hematology & Oncology, University of Alabama at Birmingham, Birmingham, AL USA; 5 Lineberger Comprehensive Cancer Center, University of North Carolina at Chapel Hill, Chapel Hill, NC USA; 6 Department of Surgery, University of Alabama at Birmingham, Birmingham, AL USA

**Keywords:** Financial hardship, Clinical trial, Breast cancer, Deep South

## Abstract

**Purpose:**

This study explored perspectives on clinical trial-related financial considerations from patients with breast cancer receiving treatment in the Deep South who declined trial participation.

**Methods:**

This qualitative content analysis included interview data from patients offered participation in a breast cancer clinical trial yet declined to participate from July 2020 to January 2024. Semi-structured interviews elucidated the influence of financial factors in the decision to decline enrollment onto a trial. Open coding was used to develop the codebook via an inductive approach to identify key concepts, patterns, and themes from the transcribed interviews. Transcribed interviews were then coded deductively by two independent reviewers.

**Results:**

Of 21 patients with breast cancer, 43% and 57% previously declined participation in a therapeutic and non-therapeutic clinical trial. Interviews revealed four themes related to the influence of financial factors in the decision to decline participation in clinical trials, including (1) trial participation expenses (e.g., direct and indirect participation expenses), (2) insurance coverage (e.g., barriers for insured, resource for uninsured), (3) socioeconomic status (inequities and trust in clinical trials), and (4) financial compensation for trial participation (e.g., facilitating participation, inappropriate incentivizing).

**Conclusion:**

Future interventions targeting financial barriers to trial participation for patients most vulnerable to experiencing financial disparities are needed to diversify cancer clinical trial participation, thus ensuring results are generalizable to all patients.

## Introduction

Participation in cancer clinical trials is low, with recent estimates of about 7% of patients with cancer having ever participated in a therapeutic clinical trial and 21% in any clinical research study [1–3]. Recent research has focused on the role of patient-level barriers to the decision to participate in a clinical trial, including medical mistrust, lack of clinical trial knowledge, issues with randomization, and concerns about efficacy or toxicity [3]. A less often cited patient-level barrier to clinical trial participation is cost [4, 5]. Our previous research of > 3500 adults found respondents with lower perceived income had double the odds of reporting a cost-related consideration as very influential to clinical trial participation than those with higher perceived income, even after accounting for patient age, race, and residence [6]. In another study of > 650 patients with cancer who had received financial assistance, 13% reported declining clinical trial participation due to cost, and of those who had participated in a clinical trial, 51% reported less willingness to participate in future clinical trials due to incurred financial hardship [7]. To increase trial participation rates, it is important to understand the specific financial factors that impact a patient’s decision to enroll on a clinical trial.

Understanding financial factors influential to the decision to participate in a clinical trial is especially important for breast cancer patients receiving care in Alabama, the fifth poorest state in the nation [8]. Alabama is one of 12 states that have not expanded Medicaid, leaving an estimated 127,000 uninsured and economically under-resourced Alabamians in the coverage gap, and thus more vulnerable to cancer-related financial hardship [9]. Almost half (44%) of Alabamians reside in rural areas, which could result in issues with transportation to receive trial treatment [10]. In our previous study, women with metastatic breast cancer receiving care at the University of Alabama at Birmingham (UAB) reported spending a mean of 155 min traveling from their home to clinic and back, resulting in a mean of 10 hours of missed work weekly for working women [11]. Thus, women with breast cancer receiving cancer care in Alabama could face inequitably high financial barriers to clinical trial participation compared to women receiving cancer care in other areas due to high poverty rates, insurance and transportation issues, and lost productivity. Therefore, this study explored perspectives on clinical trial-related financial considerations from patients with breast cancer receiving treatment in the Deep South who declined trial participation. Understanding perspectives from patients who declined enrollment due to cost could aid in designing interventions to increase clinical trial enrollment, as well as the socioeconomic diversity of participants.

## Methods

### Study design and sample

This secondary, qualitative content analysis included data on barriers and facilitators to clinical trial participation from interviews conducted from August 2023 to March 2024 at UAB, a National Cancer Institute-designated Comprehensive Cancer Center in the Deep South. For the parent study [12], eligible patients were identified through an internal database which tracks clinical trial referrals for patients with breast cancer. Eligible patients included those offered participation in either a therapeutic or non-therapeutic (e.g., behavioral) breast cancer clinical trial yet declined to participate from July 2020 to January 2024. Recruitment of eligible patients was done both in-person at clinic visits or through phone calls, with participants receiving $25 compensation for participation in the study. In accordance with the Declaration of Helsinki, this study was approved by the UAB Institutional Review Board (IRB-300001910).

### Data collection and setting

A semi-structured interview guide was developed by a surgical oncologist (LG), medical anthropologist (NH), health economist (CW), and medical students (NR, MC) based on clinical experience and practical knowledge gained through direct patient care and real-world healthcare settings. The larger interview guide included questions eliciting patient perspectives on barriers and facilitators to clinical trial participation, while this secondary analysis focuses on the influence of financial factors in their decision to decline enrollment onto a therapeutic or non-therapeutic clinical trial. Interviews were conducted by a medical student (NR) via Zoom. Patients were verbally consented before the interview began. Interviews were audio-recorded and transcribed verbatim by an independent transcription service. Patient sociodemographic information extracted from the electronic medical record included patient age, race, ethnicity, home address, marital status, religion, and insurance status. Patient home address was used to calculate miles and minutes traveled to UAB and Area Deprivation Index [13], a geographic area-level measure of neighborhood disadvantage used as a proxy measure for socioeconomic status. Previous trial participation was self-reported and abstracted from the internal clinical trial referral database.

### Data analysis

The surgical oncologist (LG) conducted an initial round of open coding to develop the codebook using an inductive approach to identify key concepts, patterns, and themes from the transcribed interviews. Transcribed interviews were then coded deductively by two independent reviewers (NR, MC), using the original open code. The independent reviewers met consistently to maintain continuity and consensus, using a third reviewer (LG) to settle any discrepancies. Intercoder reliability was assessed using the kappa statistic and found suitable (*k* = 0.73). Using iterative content analysis, prominent themes, patterns, and quotes were identified from the transcripts. All analyses were completed using NVivo V14.

## Results

### Sample characteristics

Twenty-one female patients with breast cancer were included in the study, 43% (*n* = 9) of which previously declined participation in a therapeutic clinical trial and 57% (*n* = 12) of which previously declined participation in a non-therapeutic clinical trial. The mean age of patients was 57 years (SD 12), 52% were Black or a person of color, 33% lived in a highly deprived neighborhood, and 52% were enrolled on a commercial insurance plan (Table 1). Patients traveled an average 33 miles (SD 38) from their home to UAB, which took an average of 43 min (SD 40). Half of interviewed patients (52%) had previously participated in a clinical trial. Interviews revealed four themes related to the influence of financial factors in the decision to participate in clinical trials, including (1) trial participation expenses, (2) insurance coverage, (3) socioeconomic status, and (4) financial compensation for trial participation (Table 2).

**Table 1:** Sample sociodemographic characteristics (*N* = 21)

	Overall (*N* = 21)		Patients who declined therapeutic trial (*n* = 9)		Patients who declined non-therapeutic trial (*n* = 12)	
	*n*	%	*n*	%	*n*	%
Age (mean, SD)	57 (12)		54 (12)		60 (12)	
Race						
White	10	48	6	67	4	33
Black	9	43	2	22	7	58
Asian	1	5	0	0	1	8
Decline to answer	1	5	1	11	0	0
Ethnicity						
Non-Hispanic/Latino	20	91	8	89	11	92
Unknown	2	9	1	11	1	8
Neighborhood disadvantage						
Low disadvantage	14	67	7	78	7	58
High disadvantage	7	33	2	22	5	42
Residence						
Urban	20	95	8	89	12	100
Rural	1	5	1	11	0	0
Marital status						
Married	11	52	6	67	5	42
Single	9	43	2	22	7	58
Widowed	1	5	1	11	0	0
Religious						
Yes	16	76	4	44	12	100
No	4	19	4	44	0	0
Unknown	1	5	1	11	0	0
Insurance						
Commercial	11	52	7	78	4	33
Medicaid	4	19	1	11	3	25
Medicare	7	33	1	11	6	50
Distance from patient home to UAB (mean, SD)						
Miles	33 (38)		45 (44)		24 (31)	
Minutes	43 (40)		58 (48)		31 (30)	
Previous trial participation						
Yes	11	52	5	56	6	50
No	9	43	4	44	5	42
Unknown	1	5	0	0	1	8

**Table 2:** Pertinent quotes by domain

Domain	Subdomain	Pertinent quotes
Trial participation expenses	Direct trial participation expenses	*“…I have still binders and files, we have automatic drafts coming out still for radiation…because the fees associated with triple positive breast cancer are exorbitant…”* (P18)
	Indirect trial participation expenses	*“…you’re out in a rural area, you’re going to Birmingham, you’ve got an appointment. Okay, there’s travel money, in some cases, there may be overnight clinical trials. You may have to stay in the hospital three nights at various times. You got a hotel bill, you got a gas bill. Okay, food, things like that…”* (P1)
	Trial participation expenses not a consideration	*“…No, the cost is not… I mean, you mean gas and things like that? Oh, no. No, that’s not a problem. My husband is 77, I’m 72. So it’s age and distance, time, those type things. But financially, no, that’s not an issue…”* (P5)
Insurance coverage	Barriers to trial participation for the insured	*“…If you don’t have insurance, then you would need to pay for these medical costs yourself and your insurance might not pay because it’s a clinical trial drug. So that really was pretty off-putting…”* (P7)
	Trials as a resource for the uninsured or underinsured	*“…if you don’t have insurance and you can get your treatments and your medications at no cost. That would be a really good incentive if you had something going on and you were able to get your treatments and your medications at no cost by doing it through a trial…Just say if you don’t have insurance and you want to get your condition seen about, it would be worth the long-distance travel then…”* (P11)
Socioeconomic status	Barriers related to limited economic resources	*“…I think that goes back to whether or not the person works or not. I think it goes back to the work because you have to take in consideration leave time. Whether or not they have leave. Whether or not they can afford to do this…But I think the bottom line is whether or not that person is employed or not…”* (P13)
	Clinical trials as a resource for the economically disadvantaged	*“…if I can get in a clinical trial…If someone who doesn’t have insurance or a way to cover anything over what insurance would pay and a clinical trial would get it to them paid, then yeah, they may look at it different…”* (P20)
	Socioeconomic inequities and trust in clinical trials	*“…So sometimes those in the poverty level are going to have a hard time believing they think they’re going to be taken advantage of…”* (P8)
Financial compensation for trial participation	Facilitating trial participation	*“…I think that whoever’s doing the trial should compensate them with pay so they would not lose anything although they’re being taken away from their environment and they’re having to do this… some type of compensation. It’s almost like a token of appreciation for you to do this for us…”* (P13)
	Inappropriately incentivizing trial participation	*“…. I hate to say, okay, well compensate them, but then that draws in a whole bunch of other issues because that’s just not a good thing…”* (P20)

### Trial participation expenses

Almost all patients (86%) cited both direct and indirect trial participation expenses as influential in their decision to participate. Patients also noted how trial-related expenses both hindered and facilitated trial participation.

#### Direct trial participation expenses

Direct trial participation expenses, including patient cost sharing (copayments, coinsurance, deductibles) and expenses related to the investigational agent and its side effects, were influential in the decision to participate in a clinical trial. Patients reported confusion and concern regarding whether insurance would cover trial expenses (37%) with one patient stating, *"No, I don’t think they went into details about…what would happen if they will be the one to cover it. I think they was more leaning on the fact I had insurance…I don’t remember no one going over that with me. I don’t remember that saying they were covering it or anything”* (P17). Some patients (29%) noted the intense and recurring burden of cost sharing requirements, with one stating, *“It costs money every time you walk in the door… I didn’t actually pay for Keytruda…but I had to pay co-pays every visit, and that added up”* (P4). Conversely, trials were sometimes seen as a financial relief, with patients expressing gratitude for trial sponsors covering the cost of the investigational agent (19%). One participant emphasized the financial advantage of trial participation, stating *“The trial was paid by a sponsor…there wouldn’t be anything that I would have to pay”* (P10). However, two patients highlighted the potential financial burden of managing side effects of the investigational agent as a barrier to participation, with one stating, *“And the idea was that it was just, oh, your benefit is that you’re getting this extra great drug at no cost to you. Maybe, unless I have an injury for that. Now that drug has just cost me, I mean out of control medical bills”* (P7).

#### Indirect trial participation expenses

Patients frequently cited indirect trial participation expenses such as travel, hotel, food, and parking expenses as potential barriers to participation. Travel was the most common expense identified and discussed (86%) and was noted to be more cumbersome for patients traveling long distances or requiring overnight stays. One patient described, *“You’re going to Birmingham…you’ve got travel money, a hotel bill, gas, and food…”* (P1). *“Parking gets expensive”* (P9), another patient noted, highlighting that even seemingly minor costs could accumulate over time. Similarly, another patient said, *“Well, you’re going to have your gas, which we know has gotten very high. And of course, you’ve got mileage and wear and tear on the vehicle…*” (P8). Some participants pointed out that institutional support, such as parking vouchers or reimbursement for mileage or gas, could help ease these burdens. As one patient explained, *“If they knew they could valet park for free, they wouldn’t mind coming more often”* (P16).

#### Trial participation expenses not a consideration

A minority of patients (14%) felt that trial participation expenses were not influential to the trial participation decision. One patient described that expenses were not a consideration at the time of trial enrollment because *“the money part doesn’t hit you till later, much later”* (P18). Another stated, *“But yeah, there are certain situations where I would come out of pocket and just say, I don’t care. I’m paying for it because I think it can help me live a little bit longer or whatever”* (P20).

### Insurance coverage

The role of insurance in clinical trial participation was complex, yet patients believed insurance coverage plays a large role in the decision to participate in a clinical trial.

#### Barriers to trial participation for the insured

Some patients (21%) worried their insurance would not cover medical care for the potential side effects of the trial treatment, with one patient noting, “*And so that’s why I didn’t participate because I felt like I had a lot of what ifs, what if something goes wrong, will my insurance cover it”* (P17). Another participant highlighted the lack of insurance coverage for research costs, stating, *“If you are offering someone a clinical trial but your insurance isn’t going to pay for it, then that will probably weigh on a lot of people. Being sick is expensive in this country”* (P9). Another patient noted *“if they don’t have the sponsors and insurance doesn’t cover [the trial], then that is a huge issue. I think I would be less eager to participate in it, and I would think it would affect other people’s decisions too if we had to come out of pocket…”* (P20).

#### Trials as a resource for the uninsured or underinsured

Other patients (19%) saw clinical trials as an opportunity to access treatment if uninsured, acting as a safety net. One patient stated, *“If someone who doesn’t have insurance or a way to cover anything over what insurance would pay and a clinical trial would get [the treatment] to them paid, then yeah, they may look at it different”* (P20). Finally, a few patients (19%) felt insurance coverage did not impact their decision to participate in a clinical trial. One stated, *“The social worker came in and she told me whatever my insurance didn’t cover on it, they would cover the rest”* (P19), highlighting that regardless of insurance status, she was not having to pay out of pocket.

### Socioeconomic status

#### Barriers related to limited economic resources

Individual socioeconomic status significantly shaped patient perspectives on trial participation, as patients (48%) thought trial-related expenses could act as a participation barrier for those without access to economic resources. Regarding patients lacking economic resources, one patient stated, *“Because a lot of people is poor. It got a lot of poor people than rich people. It is hard to get back and forth to the doctor”* (P12). Some patients (19%) believed trial-related expenses do not impose the same burden for those of higher socioeconomic status, making it easier for them to participate, with one patient stating, “*if you’ve got all the money in the world and all the time in the world and transportation and things like that…Probably people that have more money. I think they’re willing to jump in and take a clinical trial”* (P1). Furthermore, another patient felt expenses would not impact the decision to participate in a clinical trial for those with more economic resources, stating, *“For people who already have funds, I don’t think they would care one way or another”* (P5).

#### Clinical trials as a resource for the economically disadvantaged

Conversely, trials were sometimes seen as a resource for those with limited means, with one patient observing, *“For people who are strapped for funds, compensation or free treatment would definitely help”* (P5). Another added, *“If the clinical trial is offered with no charge, which probably some of them will do that, then they’d probably be very willing”* (P9).

#### Socioeconomic inequities and trust in clinical trials

One patient identified how social and racial dynamics linked to socioeconomic status could act as a barrier to clinical trial participation. They explained*, “You got to understand is that it’s the rich people that’s telling the lower class or the ones that’s in poverty. They’re the ones telling other African American race not to participate in these clinical trials”* (P17). This insight underscores the role of systemic distrust and perceived power imbalances in discouraging participation.

### Financial compensation for trial participation

Financial compensation emerged as a pivotal factor in patients’ decision-making processes, acting as both an incentive for participation and a practical solution to offset trial-related expenses.

#### Facilitating trial participation

Patients (86%) underscored the importance of compensation in easing financial burdens of trial participation. One participant noted, *“I think it would probably open up some more doors to people that were on the border while trying to decide if they were going to do it or not. I think that might help more people participate”* (P2). Another stated, *“But for other folks, if they can get paid $50 a visit or $100 or whatever a visit and maybe a thing for lunch probably, for coming that day. I mean, it’s sad to say, money is a motivator…for a lot of folks”* (P5). Patients were asked what amount of financial compensation would be appropriate to give research participants, and they suggested varying amounts from $50 to $700 per month. However, a common consensus among all suggestions was that the compensation should be appropriate to both cover trial-related expenses and account for their time spent participating. A patient stated, “*of course you would have to factor in for gas and parking and food for each time, and compensation on the time that they’re doing it. Because sometimes they’re not going to be able to just drive themselves, somebody else may have to drive them*” (P7). One suggested that compensation should match an average salary, stating “*You just pay someone an average salary, not a minimum wage salary. I think that’s very disrespectful to offer a minimum wage salary to someone”* (P18).

#### Inappropriately incentivizing trial participation

One patient worried financial compensation could inappropriately incentivize patients to participate, stating, “*I think you could open the door to get people that really don’t need to be in the trial study, but they’re pushing to get into it just because they would get paid. So, I think when you bring in a financial aspect as far as paying people to do things, that brings in a whole different realm of things because you could have people doing it for the wrong reasons*” (P20).

## Discussion

This study of women with breast cancer who had previously declined clinical trial enrollment found financial factors influenced their decision to not participate, which included the roles of trial participation expenses, insurance coverage, socioeconomic status, and financial compensation for trial participation. Our study is unique in two ways. First, interviews were conducted with patients who declined clinical trial enrollment, a sample traditionally difficult to recruit for other research explorations like ours. Second, our study was conducted with patients from Alabama, an environment where patients may experience multiple cost-related barriers to clinical trial participation, such as high travel burdens for patients residing in rural areas. This travel burden is exacerbated for patients living in Alabama, since recent hospital closures could result in a lack of access to trials statewide due to Alabama’s Medicaid non-expansion status, low provider reimbursement rates from Medicare and Medicaid, and high uncompensated care costs for facilities serving large proportions of patients uncovered by insurance. Results from our study can inform efforts targeting financial barriers to clinical trial participation for patients most vulnerable to experiencing financial disparities.

In our study, trial participation expenses, including both direct and indirect expenses, were influential to the consideration to participate in a clinical trial due to their recurrent nature. Though trial participants are routinely shielded from the cost of the investigational agent, as the sponsor usually covers this expense, participants are still responsible for the cost-sharing requirements of their insurance policies, including copayments, deductibles, and coinsurance for any clinic visits, telehealth consults, scans, labs, or other care received while on trial that is uncovered by the trial sponsor [14–16]. Though copayments and coinsurance are usually low-costing amounts, when recurrent, these costs could be financially burdensome for many participants. It is unclear if and how these potential costs are being communicated during the trial enrollment period, as over a third of the patients in our study reported confusion and concern regarding trial expenses during enrollment. However, it is important to discuss these expenses to lessen the risk of participants dropping out of the trial once on treatment and minimizing loss to follow-up.

Patients in our study considered financial compensation for clinical trial participation a potential avenue to increase participation in clinical trials. However, the amount of compensation individuals thought is appropriate varied, ranging from $50 to $700 per month. This estimate adds to the growing body of research in this area, with patients with cancer who had previously participated or currently participating in a clinical trial estimating needing $200–$1000 per month to compensate for trial-related out-of-pocket expenses [7, 17, 18]. It is unclear how compensation should differ by trial-related factors, such as type of trial (i.e. therapeutic or behavioral), trial phase, frequency of clinic visits, required monitoring activities, and known treatment side-effects, as well as patient-factors, such as their geographic residence, employment status, insurance status, and household income. For example, patients at our institution enrolled in an industry-sponsored clinical trial typically receive $100 per visit. Furthermore, patients with cancer enrolled on a clinical trial at a Boston-based institution and receiving financial assistance through a cancer care equity program received reimbursements that averaged $185 per month for patients living in the state of Massachusetts, $300 per month for patients living in New England, and $900 per month for patients living outside of New England [19]. Future research is needed on how to best estimate and tailor dollar amounts needed to compensate for clinical trial participation to ensure patients are not systematically excluded due to their socioeconomic status.

One patient in our study noted financial compensation could inappropriately incentivize patients to participate “*for the wrong reasons.*” The undue influence of financial incentivization could be considered an ethical concern in providing reimbursement or compensation for participation. However, previous clinical trial participants believe monetary reimbursement would serve as beneficial compensation reflecting the time, inconvenience, and risks related to participation [20, 21]. There have also been recent calls for Institutional Review Boards to consider whether payments for research participation are *high enough* to protect against patient exploitation or overburden [22]. Therefore, researchers should carefully consider a reasonable reimbursement or stipend amount for their trial to both decrease the financial burden of clinical trial participation without the unintended consequence of undue influence to participate.

The results of this study should be considered within certain limitations. This sample may not be generalizable to patients with other types of cancers or patients living outside of the Deep South. However, we consider the specificity of our study a strength. Our data may also be subject to recall bias, since patients could have been interviewed for this study up to 4 years from the time they declined clinical trial enrollment. Furthermore, though these patients declined clinical trial participation, they were still able to access and receive care at an NCI-designated Comprehensive Cancer Center. Thus, we may not have captured patients unable to access this large, urban, academic institution, who may also be the most vulnerable to financial barriers to clinical trial participation.

## Conclusion

This qualitative study of women with breast cancer receiving treatment in the Deep South who previously declined clinical trial participation revealed four themes related to the influence of financial factors in the decision to participate in clinical trials, including (1) trial participation expenses, (2) insurance coverage, (3) socioeconomic status, and (4) financial compensation for trial participation. Future research interventions targeting financial barriers to clinical trial participation for patients most vulnerable to experiencing financial disparities are needed to diversify cancer clinical trial participation, thus ensuring results are generalizable to all patients.

## Data Availability

The data that support the findings of this study are available on request from the corresponding author. The data are not publicly available due to privacy or ethical restrictions.
